# 5-(Hydroxyphenyl)-γ-Valerolactone-Sulfate, a Key Microbial Metabolite of Flavan-3-ols, Is Able to Reach the Brain: Evidence from Different in *Silico*, In Vitro and In Vivo Experimental Models

**DOI:** 10.3390/nu11112678

**Published:** 2019-11-05

**Authors:** Donato Angelino, Diogo Carregosa, Cristina Domenech-Coca, Monia Savi, Inês Figueira, Nicoletta Brindani, Saebyeol Jang, Sukla Lakshman, Aleksey Molokin, Joseph F. Urban, Cindy D. Davis, Maria Alexandra Brito, Kwang Sik Kim, Furio Brighenti, Claudio Curti, Cinta Bladé, Josep M. del Bas, Donatella Stilli, Gloria I. Solano-Aguilar, Claudia Nunes dos Santos, Daniele del Rio, Pedro Mena

**Affiliations:** 1 Human Nutrition Unit, Department of Veterinary Medicine, University of Parma, 43125 Parma, Italy; donato.angelino@unipr.it (D.A.);; 2 CEDOC, NOVA Medical School, Faculdade de Ciências Médicas, Universidade NOVA de Lisboa, 1169-056 Lisboa, Portugal; diogo.carregosa@nms.unl.pt (D.C.); claudia.nunes.santos@nms.unl.pt (C.N.d.S.); 3 Unitat de Nutrició i Salut, Centre Tecnològic de Catalunya, Eurecat, 43204 Reus, Spainjosep.delbas@eurecat.org (J.M.d.B.); 4 Department of Biochemistry and Biotechnology, Nutrigenomics Research Group, Universitat Rovira i Virgili, 43007 Tarragona, Spain; 5 Department of Chemistry, Life Sciences and Environmental Sustainability, University of Parma, 43124 Parma, Italy; monia.savi@unipr.it (M.S.); donatella.stilli@unipr.it (D.S.); 6 Instituto de Tecnologia Química e Biológica–António Xavier, Universidade Nova de Lisboa, EAN, 2781-901 Oeiras, Portugal; inesf@itqb.unl.pt; 7 Human Nutrition Unit, Department of Food and Drug, University of Parma, 43125 Parma, Italy; nicoletta.brindani@studenti.unipr.it (N.B.); furio.brighenti@unipr.it (F.B.); 8 Department of Food and Drug, University of Parma, 43125 Parma, Italy; claudio.curti@unipr.it; 9 U.S. Department of Agriculture, Northeast Area, Agricultural Research Service, Beltsville Human Nutrition Research Center, Diet Genomics and Immunology Laboratory, Beltsville, MD 20705, USA; Saebyeol.Jang@gmail.com (S.J.); Sukla.Lakshman@usda.gov (S.L.); Aleksey.Molokin@usda.gov (A.M.); Gloria.Solano-Aguilar@ars.usda.gov (G.I.S.-A.); 10 Office of Dietary Supplements, National Institutes of Health, Bethesda, MD 20892, USA; davisci@mail.nih.gov; 11 Research Institute for Medicines (iMed.ULisboa), Faculty of Pharmacy, Universidade de Lisboa, 1649-003 Lisbon, Portugal; abrito@ff.ulisboa.pt; 12 Department of Biochemistry and Human Biology, Faculty of Pharmacy, Universidade de Lisboa, 1649-003 Lisbon, Portugal; 13 Division of Infectious Diseases, Johns Hopkins University School of Medicine, Baltimore, MD 21287, USA; kwangkim@jhmi.edu; 14 Instituto de Biologia Experimental e Tecnológica, 2781-901 Oeiras, Portugal; 15 School of Advanced Studies on Food and Nutrition, University of Parma, 43124 Parma, Italy

**Keywords:** blood brain barrier, catechin, flavan-3-ol, gut, metabolites, permeability, proanthocyanidin, polyphenols, valerolactone, neurodegenerative disease

## Abstract

Phenolic compounds have been recognized as promising compounds for the prevention of chronic diseases, including neurodegenerative ones. However, phenolics like flavan-3-ols (F3O) are poorly absorbed along the gastrointestinal tract and structurally rearranged by gut microbiota, yielding smaller and more polar metabolites like phenyl-γ-valerolactones, phenylvaleric acids and their conjugates. The present work investigated the ability of F3O-derived metabolites to cross the blood-brain barrier (BBB), by linking five experimental models with increasing realism. First, an in silico study examined the physical-chemical characteristics of F3O metabolites to predict those most likely to cross the BBB. Some of these metabolites were then tested at physiological concentrations to cross the luminal and abluminal membranes of brain microvascular endothelial cells, cultured in vitro. Finally, three different in vivo studies in rats injected with pure 5-(3′,4′-dihydroxyphenyl)-γ-valerolactone, and rats and pigs fed grapes or a F3O-rich cocoa extract, respectively, confirmed the presence of 5-(hydroxyphenyl)-γ-valerolactone-sulfate (3′,4′ isomer) in the brain. This work highlighted, with different experimental models, the BBB permeability of one of the main F3O-derived metabolites. It may support the neuroprotective effects of phenolic-rich foods in the frame of the “gut-brain axis”.

## 1. Introduction

Flavan-3-ols (F3O) are among the most abundant phenolic compounds in fruits such as apples, pears, stone fruits, berries, grapes, and nuts—and in beverages, like tea and red wine, as well as in cocoa and pulses [1]. They are present in nature as simple monomers (catechins) or, more frequently, as oligomers and complex polymers (proanthocyanidins, or PACs) and, depending on the monomeric units, can be distinguished as procyanidins, prodelphinidins and propelargonidins [2]. 

Epidemiological studies have highlighted that the consumption of F3O-rich foods, in the context of a healthy diet, is positively associated with a slower rate of cognitive decline [3,4,5]. Following these findings, several dietary intervention studies have linked the consumption of F3O-rich foods to cognitive functions [6,7]. Most of these studies used dietary intake of the F3O parent compound or its glucuronidated-, sulfated or -methylated conjugates as markers of beneficial effects [8]. The use of F3O as dietary marker can be problematic in an intervention study [9]. In fact, bioavailability studies have revealed that (epi)(gallo)catechin monomers are rapidly conjugated by phase I and phase II detoxification enzymes in the gut and appear in the bloodstream from 1 h to 4 h after ingestion [10]. However, it has been estimated that less than 4% of the F3O follows this fate [11], as the majority of these phenolics reaches unmodified the colonic tract and undergoes a deep metabolism by gut microbiota [10]. As consequence of this intense structural rearrangement, several compounds characterized by lower molecular weight and higher polarity are released into the bloodstream, mainly as conjugated forms [12]. Among these, phenyl-γ-valerolactones (PVLs) and phenylvaleric acids (PVAs) have been found to reach the bloodstream from 4 h to 24 h after the F3O intake, accounting for 42% of ingested (–)-epicatechin [13]. For this reason, understanding how F3O and their conjugates and microbial derivatives can exert putative biological effects in the brain is needed to establish physiological relevance. 

A recent study hypothesized that a “gut-brain axis”, where microbially modified (poly)phenols in the gut reach the brain to activate signaling cascades to generate molecular signals that return to the gut and other target organs [14]. Nevertheless, this intriguing possibility is complicated by the unique anatomical structure of the blood-brain barrier (BBB), which is only selectively permeable to xenobiotics [15]. In fact, despite the evidence of a slight BBB permeability of (+)-catechin, (−)-epicatechin, and 5-(3′,5′-dihydroxyphenyl)-γ-valerolactone [16,17,18,19,20], there is a lack of information concerning the effective permeability of BBB by major F3O colonic metabolites such as the conjugated forms of 5-(3′,4′-dihydroxyphenyl)-γ-valerolactone, among other PVLs and PVAs [10].

**Figure 1:**
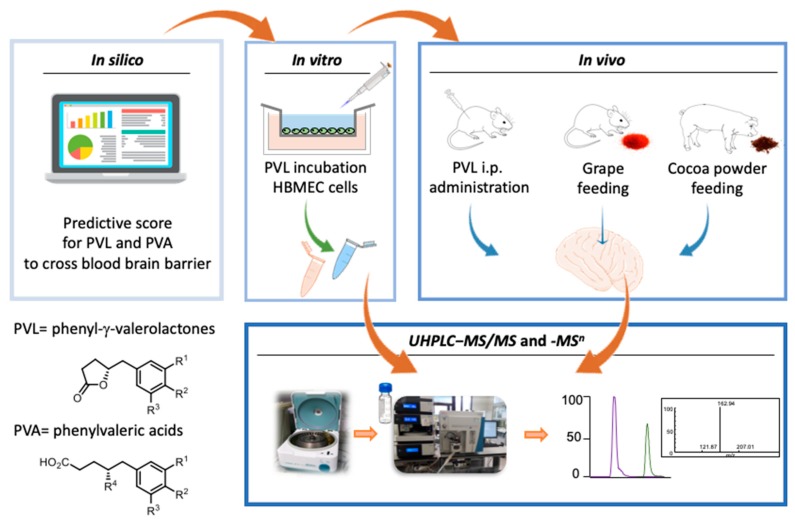
Graphical representation of the in silico, in vitro and in vivo studies conducted in order to investigate the presence of specific flavan-3-ol colonic metabolites into the brain.

In order to increase the knowledge on the putative presence of specific F3O colonic metabolites into the brain, the present manuscript reports the findings from in silico, in vitro and in vivo animal studies, with increasing level of experimental realism [21], and graphically summarized in Figure 1. Initially, an in silico approach was used to predict the compounds putatively able to cross the BBB. Then, an in vitro cell culture study was performed to evaluate the capacity of selected PVLs to cross the BBB. Finally, in vivo studies investigated the presence of PVLs and PVAs in the brain of rats and pigs treated with F3O related metabolites. One in vivo study tested the intra-peritoneal injection of pure 5-(3’,4’-dihydroxyphenyl)-γ-valerolactone in a rat model, while two others fed rats and pigs enriched sources of F3O (from lyophilized grapes and cocoa extract). 

## 2. Materials and Methods 

### 2.1. In Silico Prediction of BBB Permeability

A database containing the 67 PVL and PVA metabolites found to circulate in humans [10] was created to include their 3D structures as described below. The 3D structures were created in ChembioDraw (v.14.0, PerkinElmer, Waltham, MA, USA). Initially these structures were imported as mol files into Maestro software package (version 2018-4, Schrödinger, New York, NY, USA). The structures were then treated with LigPrep (version 2018-4, Schrödinger) using OPLS forcefield. We defined pH 7.4 ± 2.8 as biological relevant target pH, using Epik (version 2018-4, Schrödinger). For each molecule, a total of 43 molecular descriptors were calculated using QikProp (version 2018-4, Schrödinger). These molecular descriptors are relevant to predict drug-like molecules and the ones with more ability to passively permeate cellular barriers. Concerning the drug-like feature, a non-compliance score is given by #stars in agreement with previous works [22,23]. The #stars parameter indicates the number of molecular descriptors for the molecule computed by QikProp that fall outside the range of values for 95% of known drugs [22,23]. 

To create a more relevant score to predict BBB permeability, there were 12 of the 43 parameters predicted by Schrodinger software together with specific ranges for these 12 parameters found in the bibliography for BBB permeability (Supplementary Table S1). This score has been named “#BBBscore” and ranges from 0 to 12. Briefly, the twelve evaluated parameters were: molecular weight, number of donor and acceptor hydrogen bounds, Van der Waals surface area of polar atoms (PSA), the logarithm of octanol/water partition coefficient, logarithm of BBB predicted partition coefficient (QPlogBB), predicted permeability for CaCo-2 and MDCK cell lines, dipole, volume, rotatable bounds and the prediction logarithm of albumin binding (logKhsa).

### 2.2. In Vitro Transport Assay

A human brain microvascular endothelial cell (HBMEC) line was used for the in vitro model of the BBB. HBMEC line was cultured in RPMI 1640 medium (Sigma-Aldrich, St. Louis, MO, USA) supplemented with 10% fetal bovine serum, 1% non-essential amino acids, 1% minimal essential medium vitamins, 1 mM sodium pyruvate, 2mM L-glutamine (Biochrom AG, Berlin, Germany), 10% NuSerum IV (BD Biosciences, Erembodegem, Belgium), and 1% antibiotic-antimycotic solution (Sigma-Aldrich) [24]. HBMEC were plated on semi-permeable membranes (inserts) placed in cell culture wells. In this two-chamber system, where the upper and lower chambers mimic the blood and brain compartments, respectively, and the confluent HBMEC monolayer represents the BBB with the luminal and abluminal surfaces facing the “blood” and the “brain”, respectively. Cells were seeded on polyester trans-well inserts (0.4 μm, Corning Costar Corp., New York, NY, USA) at a density of 8 × 10^4^ cells/insert and treated after monolayer formation at 8 days in culture. Inserts were coated with rat-tail collagen-I (BD Biosciences, Erembodegem, Belgium) before seeding. Transport assays were conducted in Hank’s Balanced Salt Solution (HBBS) with calcium and magnesium (Gibco, Waltham, MA, USA), supplemented with 0.1% FBS as previously described [24]. Confluent monolayers of HBMEC were incubated for 2 h with 5 µM of each compound, added to the upper chamber of the culture system. Compounds, 5-(4′-hydroxyphenyl)-γ-valerolactone, 5-(3′,4′-dihydroxyphenyl)-γ-valerolactone and 5-(4′-hydroxyphenyl)-γ-valerolactone-3′-sulfate, were synthesized in house [25,26].

To assure that exposure to PVLs did not induce a disruption of barrier properties, monolayer integrity was assessed by measurement of trans-endothelial electrical resistance (TEER) using an End Ohm™ chamber coupled to an EVOMX resistance meter (World Precision Instruments, Inc., Sarasota, FL, USA), and by analysis of paracellular permeability to sodium fluorescein (Sigma-Aldrich, St. Louis, MO, USA), as described before [24]. In the end, cell media from upper and lower compartments were collected, extracted according to Sala et al. [27] but using acidified methanol (0.1% formic acid, Carlo Erba Reagents, Milan, Italy) instead of pure methanol, and frozen at −80 °C until analysis. 

### 2.3. In Vivo Rat Study with Intraperitoneal Doses of 5-(3′,4′-Dihydroxyphenyl)-γ-Valerolactone

This study was carried out in strict accordance with the recommendations in the Guide for the Care and Use of Laboratory Animals [28]. The investigation was approved by the Veterinary Animal Care and Use Committee of the University of Parma, Parma, Italy (Prot. No. 59/12) and conforms to the National Ethical Guidelines of the Italian Ministry of Health. All efforts were made to minimize animal suffering.

The study population consisted of 2 male Wistar rats (*Rattus norvegicus*) bred in our department animal facility, aged 12 weeks, weighing 385 ± 4 g (mean ± standard deviation), individually housed in a temperature-controlled room at 22–24 °C, with the light on between 7:00 AM and 7:00 PM. The bedding of the cages consisted of wood shavings, and food and water were freely available. Animals were subjected to daily intraperitoneal injection of 5-(3′,4′-dihydroxyphenyl)-γ-valerolactone, at a dose of 2 mg/kg/day, for 7 days. To prepare 5-(3′,4′-dihydroxyphenyl)-γ-valerolactone stock solution, the compound was dissolved in dimethyl sulfoxide (Sigma-Aldrich, Milan, Italy) at 0.05 mg/mL and stored in darkness at 4 °C. For each animal, immediately prior to intraperitoneal injection, an appropriate aliquot was taken from the stock solution and diluted in phosphate buffered saline (PBS) to reach the desired concentration in a final volume of 250 μL. Fifteen minutes after the last injection, animals were anaesthetized by intraperitoneal injection with 40 mg/kg ketamine chloride (Imalgene, Merial, Milan, Italy) plus 0.15 mg/kg medetomidine hydrochloride (Domitor, Pfizer Italia S.r.l., Latina, Italy). Then, the abdominal aorta was cannulated, the heart was arrested in diastole with injection of CdCl_2_ solution (100 mmol/L, iv), and the organs were perfused in retrograde with PBS to drain the blood. Brain was removed, weighed, fragmented in liquid nitrogen and stored at −80 °C until analysis.

### 2.4. In Vivo Rat Study with Grape Supplementation

The Animal Ethics Committee of the University Rovira i Virgili (Tarragona, Spain) approved all the procedures. Twenty-four 8-weeks-old male Fischer 344 rats (Charles River Laboratories, Barcelona, Spain) were housed in pairs in cages at 22 °C under two different light schedules in order to emulate season’s day length: long day photoperiod (*n* = 12, LD, 18:6h light/dark cycle) or short-day photoperiod (*n* = 12, SD, 6:18 h light/dark cycle). After 1 month under these conditions, animals in each photoperiod were orally supplemented with lyophilized red grapes [29] (100 mg per kg of body weight/day) (grape, *n* = 12) as an autumn fruit for 10 weeks or a control vehicle (control, *n* = 12). Rats were fed an *ad libitum* standard diet (2.90 kcal/g; A04, Panlab, Barcelona, Spain) and, after 14 weeks, they were deprived of food for 1 h and sacrificed by decapitation. Each brain was rapidly removed after death, flushed with cold isotonic saline buffer, weighed, frozen in liquid nitrogen and stored at −80 °C until further analysis.

### 2.5. In Vivo Pig Study with Ccocoa Powder Supplementation

All animal experiments and procedures were conducted in accordance with guidelines established and approved by the Beltsville Area Animal Care and Use Committee under protocol 12-021. The animal feeding protocol has been described elsewhere [30]. Briefly, two groups of six pigs each were randomly allocated to consume every morning, for 27 days, a 0 or 20 g/day cocoa powder supplement, with an intake of 0 and 410 mg F3O, respectively. 

The brain was removed after pigs were euthanized using Euthasol (Virbac AH, Inc., Fort Worth, TX, USA) followed by an incision of both femoral and brachial vessels for quick removal of blood. After a skin incision along the head a large craniotomy was done to expose the underlying dura followed by a large dura incision to expose the complete brain. After rinsing whole brain in phosphate buffered solution (PBS) half brain hemisphere was dissected to locate the cortex and hippocampus using a pig atlas [31]. Five mm^3^ tissue sections of cortex and hippocampus were collected in cryotubes and immediately frozen in liquid nitrogen and then kept at −80 °C until further analysis. 

### 2.6. Brain Sample Processing

Frozen brain sections were ground in liquid N_2_ to obtain the samples as a fine powder and processed as described elsewhere [32]. Briefly, approximately 1 g of each tissue sample were mixed with 1500 µL of 2% formic acid (Carlo Erba Reagents) in acetonitrile (Sigma-Aldrich, St. Louis, MO, USA) for protein precipitation (the volume for the extraction was changed proportionately to the weight of each sample). Then, samples were vortexed for 5 min, sonicated in a bath for 10 min and finally centrifuged at 16,000× *g* for 5 min. Second extraction was done using 3 mL of acidified acetonitrile. Both supernatants were pooled and reduced to dryness using a Speedvac concentrator (Thermo Savant SPD121P, Thermo Fisher Scientific Inc., San José, CA, USA). The formed pellet was suspended in 100 µL of methanol:water:formic acid (49.9:50:0.1, *v*/*v*/*v*) and centrifuged at 16,000× *g* for 5 min prior to analysis.

### 2.7. Ultra-High-Performance Liquid Chromatography−Tandem Mass Spectrometry (UHPLC−MS/MS) and –MS ^n^ Analyses 

Cell media and brain extracts were analyzed for quantification purposes by an UHPLC DIONEX Ultimate 3000 equipped with a TSQ Vantage triple quadrupole mass spectrometer (MS, Thermo Fisher Scientific Inc.), as fully reported in Brindani et al. [25]. Quantification was performed with calibration curves of standard compounds.

Targeted full MS^2^ and MS^3^ experiments were carried out to confirm the identity of some metabolites. Experiments were carried out using an Accela UHPLC 1250 equipped with a linear ion trap MS (LTQ XL) fitted with a heated electrospray ionization (ESI) probe (Thermo Fisher Scientific Inc., San José, CA, USA). Separations were performed using an Acquity UPLC HSS T3 (1.8 μm particle size) column (100 × 2.1 mm) equipped with its VanGuard pre-column (5 × 2.1 mm, 1.8 µm) (Waters, Milford, MA, USA). The volume injected was 5 µL and the column oven was set to 40 °C. Elution was performed at a flow rate of 0.5 mL/min. The gradient started with 95% of 0.1% aqueous formic and 5% of acetonitrile 0.1% formic acid for 0.5 min, followed by a 6-min linear gradient of 5% to 35% acidified acetonitrile, to reach 80% acidified acetonitrile at 7.5 min. From 7.5 to 9 min the acidified acetonitrile was kept at 80%, followed by 2 min at 5% (start conditions) to re-equilibrate the column. The MS worked in negative ionization mode, with a capillary temperature equal to 275 °C, while the source heather temperature was set to 300 °C. The sheath gas flow was 60 units, while auxiliary gas was set to 5 units, without sweep gas. The source voltage was 4.0 kV. The capillary voltage and tube lens were −33 and −98 V, respectively. Analyses were carried out using a full scan MS^2^ and MS^3^ mode, targeting specific molecular ions. Collision induced dissociation (CID) equal to 30 and 30 (arbitrary units), were used for the first and second fragmentation event, respectively. Pure helium gas was used for CID. Data processing was performed using Xcalibur software (Thermo Fisher Scientific Inc., San José, CA, USA). 

### 2.8. Statistical Analysis

Principal component analysis (PCA) of data from in silico molecular descriptors prediction with QikProp was carried out. The statistical analyses were performed using the Statistical Package for Social Science (SPSS version 23.0, SPSS Inc., Chicago, IL, USA). All relevant molecular descriptors were used for this analysis.

## 3. Results

### 3.1. In Silico Analysis 

**Figure 2:**
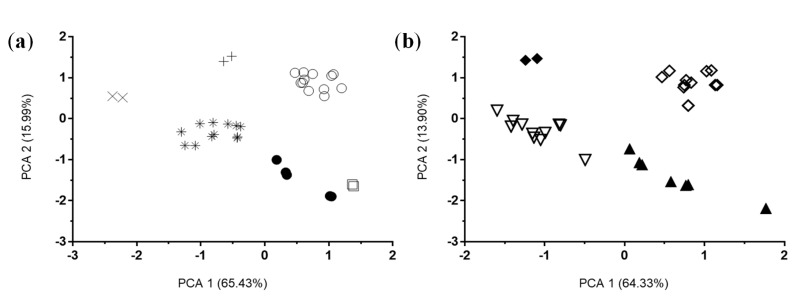
Principal component analysis of all the relevant molecular descriptors generated for the 67 molecules (PVLs and PVAs). (**a**) PCA for PVLs. Black circles: unconjugated PVLs; asterisk: PVL-sulfate conjugates (including methoxy-sulfate ones); white circles: PVL-glucuronide conjugates (including methoxy-glucuronide ones); cross: PVL-sulfate-glucuronide conjugates; oblique cross: PVL-disulfate conjugates; white squares: PVL-methoxy conjugates (not presenting sulfate or glucuronide moieties). (**b**) PCA for PVAs. Black triangles: unconjugated PVAs; white triangles: PVA-sulfate conjugates (including methoxy-sulfate ones); white rhombus: PVA-glucuronide conjugates (including methoxy-glucuronide ones); black rhombus: PVA-sulfate-glucuronide conjugates.

A total of 43 molecular descriptors of absorption, distribution, metabolism, excretion and toxicity (ADMET) were calculated for the 67 PVL and PVA metabolites described in circulation in humans [10]. Overall examination by PCA for PVL metabolites (Figure 2a) and PVA metabolites (Figure 2b) clearly clustered the metabolites by type of conjugation reflecting their similar physical-chemical properties. In addition, when sulfate or glucuronide moieties were present, additional methylation did not change the metabolite behavior.

Data showed that only 4 out of the 67 molecules comply to the range for 95% of known drugs for all 43 considered parameters: (5-(3′,4’,5’-trihydroxyphenyl)-γ-valerolactone, 5-(3′,4’-dihydroxyphenyl)-γ-valerolactone, 5-(3′,5’-dihydroxyphenyl)-γ-valerolactone and 4-hydroxy-5-phenylvaleric acid).

From the QikProp list of molecular descriptors, 24 were chosen to determine the #star parameter. From the group of evaluated molecules, 63.16% had a score of 0, meaning they were more drug-like than molecules with higher number of #stars (Supplementary Table S2). Nevertheless, the number of #stars for all molecules evaluated was inside the range of recommended values by QikProp (below 5 #stars). Interestingly, the compounds with higher number of #stars corresponded to glucuronide, sulfate-glucuronide or disulfate conjugates that showed a high hydrophilic character, as revealed by the increased number of acid groups, donor and acceptor hydrogen bonds and PSA parameters. The same conclusion for glucuronide, sulfate-glucuronide or disulfate derivatives can be made by applying Lipinski rule of five for predicting if a molecule is drug-like.

**Figure 3:**
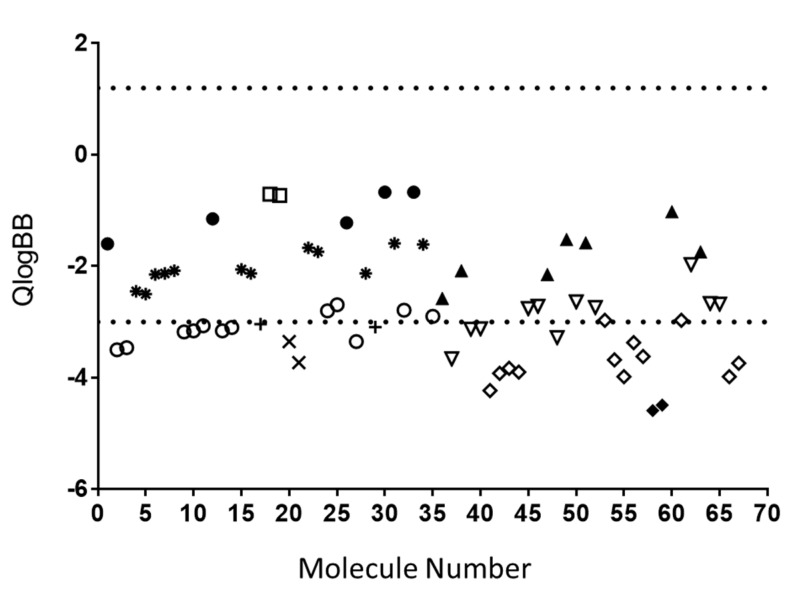
Predicted brain/blood partition coefficient (QPlogBB). QikProp predictions consider central nervous system positive molecule when QPlogBB coefficient is between –3 and 1.2 (range indicated with dashed line) for 95% of all known drugs to cross the BBB. Black circles: unconjugated PVLs; asterisk: PVL-sulfate conjugates (including methoxy-sulfate ones); white circles: PVL-glucuronide conjugates (including methoxy-glucuronide ones); cross: PVL-sulfate-glucuronide conjugates; oblique cross: PVL-disulfate conjugates; white squares: PVL-methoxy conjugates (not presenting sulfate or glucuronide moieties); black triangles: unconjugated PVAs; white triangles: PVA-sulfate conjugates (including methoxy-sulfate ones); white rhombus: PVA-glucuronide conjugates (including methoxy-glucuronide ones); black rhombus: PVA-sulfate-glucuronide conjugates.

One of the most interesting features in QikProp is the generation of molecular descriptors relevant for BBB permeation by passive diffusion. The single most relevant descriptor predicted by QikProp is QPlogBB. An analysis of this molecular descriptor revealed that 81.82% of all molecules evaluated were inside the range for 95% of known drugs to passively cross the BBB (−3.00 to 1.20) (Figure 3). Again, the majority of glucuronide, sulfate-glucuronide or disulfate conjugates were the classes of molecules out of the defined range for 95% of known drugs. 

**Figure 4:**
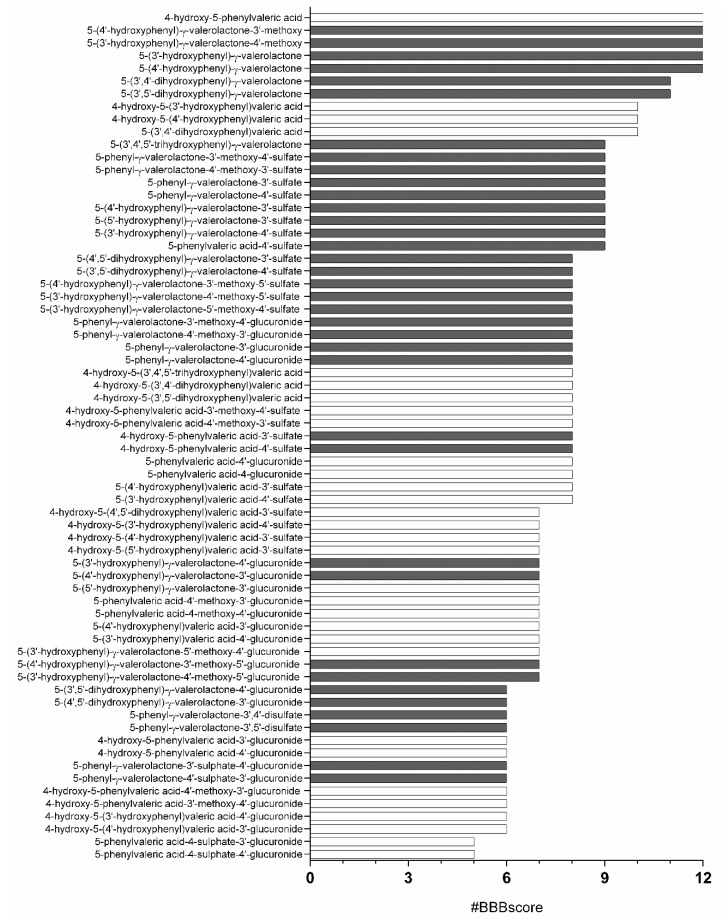
Metabolites ranked by #BBBscore. Twelve QikProp molecular descriptors specifically related with BBB permeability were used to define a score. Black bars correspond to PVLs and white bars to PVAs.

To reinforce the BBB prediction, we decided to use QPlogBB together with 11 additional molecular descriptors considered relevant in the literature for BBB permeability in the recommended ranges specifically described for blood brain permeability (Supplementary Table S1). The analysis of these 12 molecular descriptors (#BBBscore) (Supplementary Table S3) revealed 5 molecules that were inside the range for all parameters for our passive brain permeation relevant ranges: 4-hydroxy-5-phenylvaleric acid, 5-(4′-hydroxyphenyl)-γ-valerolactone-3′-methoxy, 5-(3′-hydroxyphenyl)-γ-valerolactone-4′-methoxy, 5-(4′-hydroxyphenyl)-γ-valerolactone, and 5-(3′-hydroxyphenyl)-γ-valerolactone (Figure 4).

### 3.2. Results from In Vitro and In Vivo Studies

#### 3.2.1. In Vitro Study

Three PVLs, hypothesized to be BBB permeable, i.e., 5-(4′-hydroxyphenyl)-γ-valerolactone, 5-(3′,4′-dihydroxyphenyl)-γ-valerolactone and 5-(4′-hydroxyphenyl)-γ-valerolactone-3′-sulfate were tested at 5 μM, in line with a previous report on phenolic metabolites [24], to evaluate their ability to cross from the upper to the lower layer compartments’ media of the BBB cell model using cultured-HBMEC. Moreover, the presence of newly-formed metabolites was assessed by targeted UHPLC-MS/MS analyses and considering different metabolic reactions [33], including conjugation with methyl, glucuronide, and sulfate moieties, (de)hydroxylation, and formation of their respective PVAs, as well as deconjugation in the case of 5-(4′-hydroxyphenyl)-γ-valerolactone-3′-sulfate.

5-(4′-hydroxyphenyl)-γ-valerolactone-3′-sulfate was identified and quantified in both chambers. Its concentrations ranged between 0.42 ± 0.03 μM in the upper one and 0.05 ± 0.01 μM in the lower one, with a rough estimate of a ratio 10:1 between the two compartments as well as between the incubated amount and that recorded on the luminal side of HBMEC. In the case of 5-(3′,4′-dihydroxyphenyl)-γ-valerolactone, only traces were detected in the upper compartment layer. These two metabolites did not undergo any metabolic transformation. In the case of 5-(4′-hydroxyphenyl)-γ-valerolactone, traces of the glucuronide form (5-phenyl-γ-valerolactone-4′-glucuronide) were detected the in abluminal layer.

#### 3.2.2. In Vivo Studies

**Figure 5:**
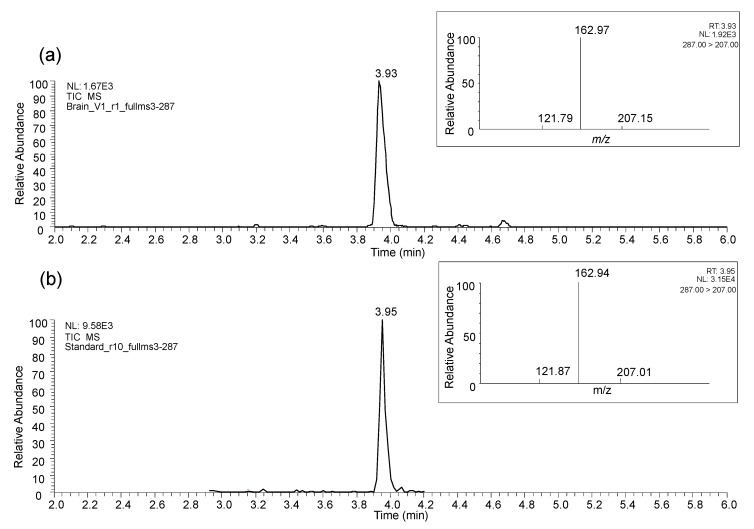
LC-MS profile 5-(hydroxyphenyl)-γ-valerolactone-sulfate (3′,4′ isomer) in a rat brain extract (**a**) and 5-(4′-hydroxyphenyl)-γ-valerolactone-3′-sulfate as reference standard (**b**). In the rectangular insets, the MS^3^ ion spectrum of 5-(hydroxyphenyl)-γ-valerolactone-sulfate (**a**) and the reference compound (**b**).

The intraperitoneal administration of 5-(3′,4′-dihydroxyphenyl)-γ-valerolactone into rats led to the detection in brain of the sulfate form of 5-(3′,4′-dihydroxyphenyl)-γ-valerolactone ([M-H]^−^ *m/z* 287) (Figure 5a), while the aglycone form administered was not detected. The identification was confirmed with specific full MS^2^ and full MS^3^ experiments and by comparison with the reference available compound, 5-(4′-hydroxyphenyl)-γ-valerolactone-3′-sulfate (Figure 5b). 

Unfortunately, it was not possible to reveal which one of the two known isomers, 5-(3′-hydroxyphenyl)-γ-valerolactone-4′-sulfate or 5-(4′-hydroxyphenyl)-γ-valerolactone-3′-sulfate, was present in the brain tissue due to their co-elution under the chromatographic conditions used and their identical fragmentation patterns [25]. For this reason, we might assume that the identification is the sum of the two isomers. Of note, the compound was detected in the brain of the two animals used.

In both rat and pig animal models supplemented with food products, targeted analysis of main circulating PVLs and PVAs in brain tissues revealed the putative presence of some PVL metabolites in some samples, the majority of them presenting peak areas very close to the limit of detection (LOD) estimated for the standards available (data not shown). Only one PVL was unequivocally found in the samples of rat and pig brain cortex. Again, the compound was identified as the sulfate form of 5-(3′,4′-dihydroxyphenyl)-γ-valerolactone, confirmed with specific full MS^2^ and full MS^3^ experiments and by comparison with 5-(4′-hydroxyphenyl)-γ-valerolactone-3′-sulfate, as reported before. It was identified in most of the samples but peak areas were mainly below the lower limit of quantification (LLOQ), which made quantification not feasible.

In the rats fed the grape supplementation, 5-(hydroxyphenyl)-*γ*-valerolactone-sulfate (3′,4′ isomer) was detected in control and treated samples (33% of samples for both conditions), regardless of the exposure to different light schedules (LD or SD). In pigs fed cocoa extract, 5-(hydroxyphenyl)-γ-valerolactone-sulfate was identified only in the brain cortex of those fed 20 g/day cocoa powder animals (100% of the samples), and not in the control group.

## 4. Discussion

Several observational and intervention studies suggested that diets rich in (poly)phenols beneficially affect nervous system functions [34]. However, their scarce bioavailability and structural modifications along the gastrointestinal tract make a sound conclusion very hard to draw. Despite some literature that supports a minimal capacity of some phenolics and some known colonic metabolites to cross the BBB, there is still a lack of information regarding the behavior of the major colonic metabolites derived from F3O in the brain. The present work used an in silico approach to predict the potential BBB permeability of numerous colonic metabolites, specifically formed from F3O. Results confirmed findings of a previous study [24], where sulfation and methylation processes had been found to improve the BBB permeability of small phenolic acids. In addition, unconjugated monohydroxylated and dihydroxylated PVLs have also been found to have a higher #BBB score, possibly because of their small steric hindrance and higher polarity.

Compounds selected for in vitro testing were based on the #BBB score found by the in silico analysis, their appearance at relevant concentrations into the bloodstream and the availability of synthesized standards. To do this, we used 5 μM concentration of the metabolites in well, which is sensibly higher than the concentrations reported in circulation, but supportive for the study of their putative BBB crossing. This resulted in the choice of one monohydroxyPVL (5-(4′-hydroxyphenyl)-γ-valerolactone), a dihydroxyPVL (5-(3′,4′-dihydroxyphenyl)-γ-valerolactone) and a sulfate form of the dihydroxyPVL (5-(4′-hydroxyphenyl)-γ-valerolactone-3′-sulfate), to better understand the impact of molecular structure on the BBB permeability. While no traces of 5-(4′-hydroxyphenyl)-γ-valerolactone and 5-(3′,4′-dihydroxyphenyl)-γ-valerolactone were found in either the apical or basal layers of HBMEC after 2 h treatment, 5-(4′-hydroxyphenyl)-γ-valerolactone-3′-sulfate was notably detected. Unno et al. [17] incubated in a BBB model consisting in co-cultures of endothelial cells, perycytes and astrocytes 30 μM of 5-(3′,5’-dihydroxyphenyl)-γ-valerolactone and its sulfate and glucuronide conjugates, for 30 min. Results showed a roughly 3:100 ratio between the luminal and abluminal concentrations for the three compounds, compared to 10:1 in this study for 5-(4′-hydroxyphenyl)-γ-valerolactone-3′-sulfate. However, luminal and abluminal concentrations of metabolites were almost 5-fold higher and 2-fold lower, respectively, to the one found in our study. Those differences might be ascribed to the higher concentrations of metabolites and the lower incubation time used, despite both conjugated and unconjugated metabolites were found to permeate the BBB [17]. To our knowledge, this was the only study investigating the BBB permeability of some PVLs to date. On the other hand, the identification of the glucuronide form of 5-(4′-hydroxyphenyl)-γ-valerolactone emphasised the need to further study cell metabolism when performing cell culture studies [35]. The absence of metabolic transformations by HBMEC cells to 5-(4′-hydroxyphenyl)-γ-valerolactone-3′-sulfate, which has been reported to be extensively modified by T24 bladder epithelial cells [33], also accounts for the cell type-specificity of some metabolic reactions.

To confirm the in silico and in vitro results, different animal models were used with an increasing complexity in the design. Both our rat and pig in vivo studies confirmed the results from the in silico and cell culture models results, as 5-(hydroxyphenyl)-γ-valerolactone-sulfate (3′,4′ isomer) was detected in brain tissues after treatment of the animals. This metabolite has been identified in brain tissues of both rat groups exposed to the SD and LD photoperiod. This finding suggested that the exposure to different light/dark cycles does not appear to affect the BBB permeability of this specific metabolite, but further studies are needed in order to confirm this statement. Moreover, no speculation can be done for other metabolites until further studies are completed. The detection of the metabolite in both treated and control animals may be related to the ubiquitous presence of F3O in animal chows, which are mainly composed of PAC containing plant-based ingredients. On the other hand, the fact that a major circulating PVL, 5-(4′-hydroxyphenyl)-y-valerolactone-3′-glucuronide [36], was not found in any sample is supported by the low #BBB score found by the in silico analysis.

Interestingly, 5-(hydroxyphenyl)-γ-valerolactone-sulfate identification in brains of pigs fed a cocoa powder supplemented diet is in line with the results of previous work in pigs using the same animal model and study protocol [30]. In fact, the presence of 5-(hydroxyphenyl)-γ-valerolactone-sulfate was detected in urine samples of pigs fed 0 to 20 g cocoa powder/day [30]. Although this compound was detected in significantly higher concentrations in samples of animals treated with cocoa powder, its presence in control samples was problematic. The presence of F3O in the chow of the control animals, as also confirmed by the presence of some phenolic acids derived from the same F3O metabolism [30], is in line with the aforementioned rat study.

The identification of the same PVL metabolite in brain tissues, regardless of the delivery of PVL precursors via oral or intraperitoneal routes and two animal species is the major strength of this study. To the best of the authors’ knowledge, this is the first study demonstrating that a 5-(hydroxyphenyl)-γ-valerolactone-sulfate is able to cross the BBB in animal models. It is also worth noting that this compound is one of the main PVLs in circulation [10]. On the contrary, the presence of this unique compound and the lack of other PVLs or PVAs are the main analytical and physiological limitations of this study. Concerning the analytical chemistry of these compounds, they generally show poor ionization and, consequently, ESI-MS^n^ analysis is characterized by relatively high LODs/LLOQs [25]. This unfavorable analytical feature for some PVLs and PVAs may hide the nature of the exact pool of F3O microbial metabolites able to cross the BBB. On the other hand, the absence in brain of significant levels F3O metabolites in the brain may be related to the transient passage of phenolic metabolites through the BBB, and their consequent lack of accumulation in the brain tissue [14]. 

## 5. Conclusions

The present work explores how the physical-chemical characteristics of the major F3O colonic metabolites affect the BBB permeability, and whether these predictions can be supported by models in in vitro and in vivo. The results clearly demonstrated the permeability of the BBB towards a specific F3O colonic metabolite, namely 5-(hydroxyphenyl)-γ-valerolactone-sulfate. 

Further research is needed to better understand the passage and biological effect of F3O metabolites in brain tissues, by considering (i) the exact isomer(s) occurring at brain level (position of the sulfate in the phenolic ring -3′ or 4′- and *R*- or *S*-enantiomers); (ii) the inter-individual variability in the metabolism of F3O, which may lead to significant differences in the production of such colonic metabolites; (iii) the mechanisms at the BBB level that regulate the permeability towards F3O colonic metabolites; and, in turn, (iv) their bioactivity. These studies are required in order to support the epidemiological literature addressing the neuroprotective effects attributed to (poly)phenol-rich foods in the context of the purported “gut-brain axis”.

## Supplementary Materials

The following are available online at https://www.mdpi.com/2072-6643/11/11/2678/s1, Table S1: List of molecular descriptors relevant, based upon the literature, for #BBBscore together with the respective range, Table S2: Number of #stars defined by QikProp for all molecules evaluated, Table S3: Predicted values for the 12 molecular descriptors selected for evaluation in the #BBBscore for all molecules.
