# Supplementary material for: 5-(Hydroxyphenyl)-γ-Valerolactone-Sulfate, a Key Microbial Metabolite of Flavan-3-ols, Is Able to Reach the Brain: Evidence from Different in Silico, In Vitro and In Vivo Experimental Models
Source: Nutrients. 2019 Nov 5;11(11):2678. doi: 10.3390/nu11112678 (PMC6893823; doi:10.3390/nu11112678)
Supplement: Supplementary file 1 [file nutrients-11-02678-s001.pdf]

Table S1. List of molecular descriptors relevant, based upon the literature, for #BBBscore together with the respective range.

Molecular Descriptors	Range for BBB	Comments	Reference
MW	< 450 g/mol	Molecular weight. Compounds with high molecular weight are unable to passively cross the blood brain barrier. This can be related with volume.	van de Waterbeemd et al. 1997 [1]
Dipole	1–12.5	Predicted dipole moment for each molecule.	Figueira et al. 2017 [2]
volume	500–200 A^3	Predicted volume for each molecule.	Figueira et al. 2017 [2]
Donor HB	0	Number of hydrogen bound donors. No hydrogen bond donors facilitates their desolvation before entering the lipophilic phase of the cell membranes.	Eigenmann et al. 2016 [3]
Acceptor HB	≤5	Number of acceptor hydrogen bonds. Low effective number of H-bond acceptors (<5) facilitates their desolvation before entering the lipophilic phase of the cell membranes.	Eigenmann et al. 2016 [3]
QLogPo/w	4	Logarithm of the partition coefficient between octanol and water.	Kelder et al. 1999 [4]
QLogBB	−3–1.2	The QikProp model for brain/blood partitioning predicts favorable blood brain barrier passive permeation for molecules which range between -3.0 and 1.2 (based upon 95% of known drugs).	Kelder et al. 1999 [4]
QPPcaco	≥500 nm/s	QikProp predicted CACO-2 cell line permeability in nm/s. Above the recommended is consider great based upon 95% of known drugs.	Eigenmann et al. 2016 [3]
QPPMDCK	≥500 nm/s	QikProp predicted MDCK cell line permeability in nm/s. Above the recommended is consider great based upon 95% of known drugs.	Eigenmann et al. 2016 [3]
QPlogKhsa	−1.5–1.5	Prediction of binding to serum albumin.	Figueira et al. 2017 [2]
PSA	70–90	Polar surface area (PSA). Recommended below the thresholds of 90 Å2 (ref. a) and 70 Å2 (ref-b).	a) van de Waterbeemd et al. 1997 [1]; b) Kelder et al. 1999 [4]
Rotatable bonds	≤6	Number of rotatable bonds. Less than 6 bonds recommended for CNS drugs.	Brito-Sánchez et al. 2015 [5]

References

1. van de Waterbeemd, H.; Camenisch, G.; Folkers, G.; Chretien, J.R.; Raevsky, O.A. Estimation of Blood-Brain Barrier Crossing of Drugs Using Molecular Size and Shape, and H-Bonding Descriptors. *J. Drug Target.* **1998**, *6*, 151–165.
2. Figueira, I.; Garcia, G.; Pimpão, R.C.; Terrasso, A.P.; Costa, I.; Almeida, A.F.; Tavares, L.; Pais, T.F.; Pinto, P.; Ventura, M.R.; et al. Polyphenols journey through blood-brain barrier towards neuronal protection. *Sci. Rep.* **2017**, *7*, 11456.
3. Eigenmann, D.E.; Dürig, C.; Jähne, E.A.; Smieško, M.; Culot, M.; Gosselet, F.; Cecchelli, R.; Helms, H.C.C.; Brodin, B.; Wimmer, L.; et al. In vitro blood–brain barrier permeability predictions for GABA-A receptor modulating piperine analogs. *Eur. J. Pharm. Biopharm.* **2016**, *103*, 118–126.

4. Kelder, J.; Grootenhuis, P.D.; Bayada, D.M.; Delbressine, L.P.; Ploemen, J.P. Polar molecular surface as a dominating determinant for oral absorption and brain penetration of drugs. *Pharm. Res.* **1999**, *16*, 1514–9.
5. Brito-Sánchez, Y.; Marrero-Ponce, Y.; Barigye, S.J.; Yaber-Goenaga, I.; Morell Pérez, C.; Le-Thi-Thu, H.; Cherkasov, A. Towards Better BBB Passage Prediction Using an Extensive and Curated Data Set. *Mol. Inform.* **2015**, *34*, 308–330.

Table S2. Number of #stars defined by QikProp for all molecules evaluated.

Molecule	#Stars
4-Hydroxy-5-phenylvaleric acid	0
5-(4-Hydroxyphenyl)-γ-valerolactone-3-methoxy	0
5-(3-Hydroxyphenyl)-γ-valerolactone-4-methoxy	0
5-(3′-Hydroxyphenyl)-γ-valerolactone	0
5-(4′-Hydroxyphenyl)-γ-valerolactone	0
5-(3′,4′-Dihydroxyphenyl)-γ-valerolactone	0
5-(3′,5′-Dihydroxyphenyl)-γ-valerolactone	0
4-Hydroxy-5-(3′-hydroxyphenyl)valeric acid	0
4-Hydroxy-5-(4′-hydroxyphenyl)valeric acid	0
5-(3′,4′-Dihydroxyphenyl)valeric acid	0
5-(3′,4′,5′-Trihydroxyphenyl)-γ-valerolactone	0
5-Phenyl-γ-valerolactone-4′-methoxy-3′-sulfate	0
5-Phenyl-γ-valerolactone-3′-methoxy-4′-sulfate	0
5-Phenyl-γ-valerolactone-3′-sulfate	0
5-(4′-Hydroxyphenyl)-γ-valerolactone-3′-sulfate	0
5-(3′-Hydroxyphenyl)-γ-valerolactone-4′-sulfate	0
5-(5′-Hydroxyphenyl)-γ-valerolactone-3′-sulfate	0
5-Phenyl-γ-valerolactone-4′-sulfate	0
5-Phenylvaleric acid-4-sulfate	0
5-(4′,5′-Dihydroxyphenyl)-γ-valerolactone-3′-sulfate	0
5-(3′,5′-Dihydroxyphenyl)-γ-valerolactone-4′-sulfate	0
5-(4′-Hydroxyphenyl)-γ-valerolactone-3-methoxy-5-sulfate	0
5-(3′-Hydroxyphenyl)-γ-valerolactone-4-methoxy-5-sulfate	0
5-(3′-Hydroxyphenyl)-γ-valerolactone-5-methoxy-4-sulfate	0
5-Phenyl-γ-valerolactone-3′-methoxy-4′-glucuronide	0
5-Phenyl-γ-valerolactone-4′-methoxy-3′-glucuronide	0
5-Phenyl-γ-valerolactone-3′-glucuronide	0
5-Phenyl-γ-valerolactone-4′-glucuronide	0
4-Hydroxy-5-(3′,4′,5′-trihydroxyphenyl)valeric acid	0
4-Hydroxy-5-(3′,4′-dihydroxyphenyl)valeric acid	0
4-Hydroxy-5-(3′,5′-dihydroxyphenyl)valeric acid	0
4-Hydroxy-5-phenylvaleric acid-3-methoxy-4-sulfate	0
4-Hydroxy-5-phenylvaleric acid-4-methoxy-3-sulfate	0
4-Hydroxy-5-phenylvaleric acid-3′-sulfate	0
4-Hydroxy-5-phenylvaleric acid-4′-sulfate	0
5-Phenylvaleric acid-4-glucuronide	0
5-Phenylvaleric acid-4-glucuronide	0
5-(4′-Hydroxyphenyl)valeric acid-3′-sulfate	0
5-(3′-Hydroxyphenyl)valeric acid-4′-sulfate	0
5-(4′-Hydroxyphenyl)-γ-valerolactone-3′-glucuronide	2
5-(3′-Hydroxyphenyl)-γ-valerolactone-4′-glucuronide	2
4-Hydroxy-5-(3′-hydroxyphenyl)valeric acid-4′-sulfate	1
4-Hydroxy-5-(4′-hydroxyphenyl)valeric acid-3′-sulfate	1

5-(3′-Hydroxyphenyl)-γ-valerolactone-5-methoxy-4-glucuronide	2
5-(5′-Hydroxyphenyl)-γ-valerolactone-3′-glucuronide	3
4-Hydroxy-5-(4′,5′-dihydroxyphenyl)valeric acid-3′-sulfate	2
4-Hydroxy-5-(5′-hydroxyphenyl)valeric acid-3′-sulfate	2
5-Phenylvaleric acid-4-methoxy-3′-glucuronide	2
5-Phenylvaleric acid-4-methoxy-4′-glucuronide	2
5-(4′-Hydroxyphenyl)valeric acid-3-glucuronide	3
5-(3′-Hydroxyphenyl)valeric acid-4-glucuronide	2
5-(4′-Hydroxyphenyl)-γ-valerolactone-3-methoxy-5-glucuronide	3
5-(3′-Hydroxyphenyl)-γ-valerolactone-4-methoxy-5-glucuronide	3
5-(4′,5′-Dihydroxyphenyl)-γ-valerolactone-3′-glucuronide	3
5-(3′,5′-Dihydroxyphenyl)-γ-valerolactone-4′-glucuronide	3
5-Phenyl-γ-valerolactone-3′,4′-disulfate	3
5-Phenyl-γ-valerolactone-3′,5′-disulfate	3
4-Hydroxy-5-phenylvaleric acid-3′-methoxy-4′-glucuronide	3
4-Hydroxy-5-phenylvaleric acid-methoxy-glucuronide	3
4-Hydroxy-5-phenylvaleric acid-3′-glucuronide	3
4-Hydroxy-5-phenylvaleric acid-4′-glucuronide	3
4-Hydroxy-5-(3′-hydroxyphenyl)valeric acid-4′-glucuronide	4
5-Phenyl-γ-valerolactone-3-sulfate-4-glucuronide	3
5-Phenyl-γ-valerolactone-4-sulfate-3-glucuronide	3
4-Hydroxy-5-(4′-hydroxyphenyl)valeric acid-3′-glucuronide	4
5-Phenylvaleric acid-4-sulfate-3′-glucuronide	4
5-Phenylvaleric acid-4-sulfate-4′-glucuronide	4

Table S3. Predicted values for the 12 molecular descriptors selected for evaluation in the #BBBscore for all molecules.

Molecule	MW	dipole	volume	donorHB	accptHB	QPlogPo/w	QPPCaco	QPlogBB	QPPMDCK	QPlogKhsa	PSA	#rotor
5-(3′,4′,5′-Trihydroxyphenyl)-γ-valerolactone	224.21	5.08	721.13	3.00	5.25	0.04	84.76	−1.60	34.34	−0.56	107.28	5
5-(4′,5′-Dihydroxyphenyl)-γ-valerolactone-3′-glucuronide	400.34	7.36	1086.79	6.00	12.10	−1.20	0.60	−3.50	0.21	−0.97	219.33	9
5-(3′,5′-Dihydroxyphenyl)-γ-valerolactone-4′-glucuronide	400.34	7.35	1094.81	6.00	12.10	−1.07	0.83	−3.46	0.29	−0.99	214.44	9
5-(4′,5′-Dihydroxyphenyl)-γ-valerolactone-3′-sulfate	304.27	7.04	845.57	3.00	9.00	−0.52	4.12	−2.45	1.71	−0.99	154.58	7
5-(3′,5′-Dihydroxyphenyl)-γ-valerolactone-4′-sulfate	304.27	5.75	850.14	3.00	9.00	−0.48	4.47	−2.50	1.86	−1.00	151.96	7
5-(4′-Hydroxyphenyl)-γ-valerolactone-3-methoxy-5-sulfate	318.30	5.99	908.04	2.00	9.00	0.16	11.32	−2.15	5.10	−0.95	140.13	7
5-(3′-Hydroxyphenyl)-γ-valerolactone-4-methoxy-5-sulfate	318.30	4.93	905.09	2.00	9.00	0.15	10.89	−2.13	4.90	−0.95	140.17	7
5-(3′-Hydroxyphenyl)-γ-valerolactone-5-methoxy-4-sulfate	318.30	5.39	908.29	2.00	9.00	0.21	12.68	−2.08	5.75	−0.94	137.48	7
5-(4′-Hydroxyphenyl)-γ-valerolactone-3-methoxy-5-glucuronide	414.37	7.93	1137.98	5.00	12.10	−0.56	1.37	−3.18	0.51	−0.90	204.78	9
5-(3′-Hydroxyphenyl)-γ-valerolactone-4-methoxy-5-glucuronide	414.37	8.24	1155.79	5.00	12.10	−0.42	1.83	−3.16	0.69	−0.89	204.38	9
5-(3′-Hydroxyphenyl)-γ-valerolactone-5-methoxy-4-glucuronide	414.37	7.46	1149.48	5.00	12.10	−0.39	2.09	−3.07	0.80	−0.90	200.09	9
5-(3′,4′-Dihydroxyphenyl)-γ-valerolactone	208.21	6.75	707.23	2.00	4.50	0.72	235.74	−1.15	103.76	−0.44	85.50	4
5-(4′-Hydroxyphenyl)-γ-valerolactone-3′-glucuronide	384.34	9.02	1080.62	5.00	11.35	−0.59	1.42	−3.16	0.53	−0.89	197.35	8
5-(3′-Hydroxyphenyl)-γ-valerolactone-4′-glucuronide	384.34	7.61	1073.63	5.00	11.35	−0.60	1.57	−3.10	0.59	−0.90	197.69	8

5-(4'-Hydroxyphenyl)-γ-valerolactone-3'-sulfate	288.27	5.33	830.74	2.00	8.25	0.03	10.41	-2.06	4.66	-0.95	132.83	6
5-(3′-Hydroxyphenyl)-γ-valerolactone-4′-sulfate	288.27	4.52	836.46	2.00	8.25	0.04	9.68	-2.13	4.30	-0.94	132.72	6
5-Phenyl-γ-valerolactone-3′,4′-disulfate	368.33	11.11	964.26	2.00	12.00	-0.66	0.48	-3.04	0.22	-1.52	181.82	8
5-(4-Hydroxyphenyl)-γ-valerolactone-3-methoxy	222.24	6.79	763.72	1.00	4.50	1.51	736.05	-0.71	355.22	-0.30	71.15	4
5-(3-Hydroxyphenyl)-γ-valerolactone-4-methoxy	222.24	7.09	763.30	1.00	4.50	1.50	712.55	-0.73	342.97	-0.30	71.43	4
5-Phenyl-γ-valerolactone-3-sulfate-4-glucuronide	464.40	6.73	1161.54	5.00	15.10	-1.03	0.20	-3.35	0.08	-1.33	242.31	10
5-Phenyl-γ-valerolactone-4-sulfate-3-glucuronide	464.40	6.49	1183.24	5.00	15.10	-1.14	0.12	-3.73	0.05	-1.36	243.85	10
5-Phenyl-γ-valerolactone-4'-methoxy-3'-sulfate	302.30	5.33	895.80	1.00	8.25	0.73	29.36	-1.67	14.29	-0.91	118.94	6
5-Phenyl-γ-valerolactone-3'-methoxy-4'-sulfate	302.30	5.40	895.79	1.00	8.25	0.68	25.98	-1.74	12.52	-0.92	118.93	6
5-Phenyl-γ-valerolactone-3'-methoxy-4'-glucuronide	398.37	3.98	1133.02	4.00	11.35	0.04	3.16	-2.80	1.25	-0.79	184.48	8
5-Phenyl-γ-valerolactone-4'-methoxy-3'-glucuronide	398.37	8.08	1121.75	4.00	11.35	0.02	3.41	-2.69	1.36	-0.79	184.94	8
5-(3',5'-Dihydroxyphenyl)-γ-valerolactone	208.21	6.98	708.49	2.00	4.50	0.66	200.78	-1.22	87.23	-0.44	86.39	4
5-(5'-Hydroxyphenyl)-γ-valerolactone-3'-glucuronide	384.34	8.16	1089.69	5.00	11.35	-0.68	0.96	-3.35	0.34	-0.88	201.29	8
5-(5'-Hydroxyphenyl)-γ-valerolactone-3'-sulfate	288.27	8.94	821.14	2.00	8.25	-0.12	7.66	-2.13	3.34	-0.95	135.27	6
5-Phenyl-γ-valerolactone-3′,5′-disulfate	368.33	10.10	968.14	2.00	12.00	-0.70	0.39	-3.09	0.18	-1.50	183.38	8
5-(3'-Hydroxyphenyl)-γ-valerolactone	192.21	6.09	685.70	1.00	3.75	1.35	661.91	-0.67	316.70	-0.32	63.83	3
5-Phenyl-γ-valerolactone-3′-sulfate	272.27	8.19	816.02	1.00	7.50	0.59	27.03	-1.59	13.11	-0.91	112.28	5

5-Phenyl-γ-valerolactone-3'-glucuronide	368.34	4.87	1060.33	4.00	10.60	-0.10	2.81	-2.79	1.10	-0.79	179.50	7
5-(4'-Hydroxyphenyl)-γ-valerolactone	192.21	5.82	685.34	1.00	3.75	1.35	662.89	-0.67	317.21	-0.33	63.82	3
5-phenyl-γ-valerolactone-4′-sulfate	272.27	8.28	804.60	1.00	7.50	0.48	24.25	-1.61	11.65	-0.94	112.22	5
5-Phenyl-γ-valerolactone-4'-glucuronide	368.34	10.53	1069.96	4.00	10.60	-0.09	2.60	-2.90	1.01	-0.80	179.12	7
4-Hydroxy-5-(3',4',5'-trihydroxyphenyl)valeric acid	242.23	5.60	784.08	5.00	5.95	-0.14	4.63	-2.58	1.89	-0.96	136.41	9
4-Hydroxy-5-(4',5'-dihydroxyphenyl)valeric acid-3'-sulfate	322.29	3.94	913.32	5.00	9.70	-0.67	0.19	-3.66	0.08	-1.32	183.55	11
4-Hydroxy-5-(3',4'-dihydroxyphenyl)valeric acid	226.23	4.90	762.79	4.00	5.20	0.47	12.76	-2.08	5.64	-0.83	115.00	8
4-Hydroxy-5-(3'-hydroxyphenyl)valeric acid-4'-sulfate	306.29	4.63	891.96	4.00	8.95	-0.06	0.52	-3.13	0.23	-1.22	162.25	10
4-Hydroxy-5-(4'-hydroxyphenyl)valeric acid-3'-sulfate	306.29	3.10	891.91	4.00	8.95	-0.06	0.53	-3.12	0.24	-1.22	162.17	10
4-Hydroxy-5-(3'-hydroxyphenyl)valeric acid-4'-glucuronide	402.35	6.64	1139.13	7.00	12.05	-0.77	0.08	-4.23	0.03	-1.27	226.48	12
4-Hydroxy-5-(4'-hydroxyphenyl)valeric acid-3'-glucuronide	402.35	5.59	1128.67	7.00	12.05	-0.65	0.12	-3.92	0.05	-1.25	226.18	12
4-Hydroxy-5-phenylvaleric acid-3'-methoxy-4'-glucuronide	416.38	8.78	1197.71	6.00	12.05	-0.05	0.19	-3.83	0.08	-1.14	213.06	12
4-Hydroxy-5-phenylvaleric acid-methoxy-glucuronide	416.38	7.39	1199.07	6.00	12.05	-0.09	0.17	-3.90	0.07	-1.14	213.22	12
4-Hydroxy-5-phenylvaleric acid-3-methoxy-4-sulfate	320.31	4.90	951.90	3.00	8.95	0.65	1.36	-2.76	0.66	-1.12	148.36	10
4-Hydroxy-5-phenylvaleric acid-4-methoxy-3-sulfate	320.31	5.25	951.29	3.00	8.95	0.67	1.43	-2.72	0.69	-1.12	148.13	10
4-Hydroxy-5-(3',5'-dihydroxyphenyl)valeric acid	226.23	5.04	764.06	4.00	5.20	0.42	10.87	-2.15	4.74	-0.83	115.96	8
4-Hydroxy-5-(5'-hydroxyphenyl)valeric acid-3'-sulfate	306.29	5.69	896.19	4.00	8.95	-0.15	0.38	-3.28	0.17	-1.22	164.15	10

4-Hydroxy-5-(3'-hydroxyphenyl)valeric acid	210.23	3.56	738.77	3.00	4.45	1.18	40.42	-1.52	19.63	-0.70	92.69	7
4-Hydroxy-5-phenylvaleric acid-3'-sulfate	290.29	7.85	872.60	3.00	8.20	0.53	1.30	-2.64	0.62	-1.13	141.52	9
4-Hydroxy-5-(4'-hydroxyphenyl)valeric acid	210.23	4.08	740.63	3.00	4.45	1.16	36.01	-1.58	17.32	-0.70	93.29	7
4-Hydroxy-5-phenylvaleric acid-4'-sulfate	290.29	6.31	867.93	3.00	8.20	0.41	1.00	-2.74	0.47	-1.13	142.50	9
5-Phenylvaleric acid-4-glucuronide	370.36	6.48	1108.77	4.00	11.50	0.49	0.81	-2.97	0.37	-1.14	176.72	11
4-Hydroxy-5-phenylvaleric acid-3'-glucuronide	386.36	8.53	1117.19	6.00	11.30	-0.19	0.18	-3.68	0.07	-1.13	208.60	11
4-Hydroxy-5-phenylvaleric acid-4'-glucuronide	386.36	6.61	1124.81	6.00	11.30	-0.30	0.13	-3.98	0.05	-1.16	208.71	11
5-Phenylvaleric acid-4-methoxy-3'-glucuronide	400.38	7.67	1169.69	5.00	11.30	0.45	0.37	-3.37	0.16	-1.02	195.53	11
5-Phenylvaleric acid-4-methoxy-4'-glucuronide	400.38	3.33	1176.97	5.00	11.30	0.37	0.30	-3.62	0.13	-1.04	194.69	11
5-Phenylvaleric acid-4-sulfate-3'-glucuronide	466.41	5.10	1256.35	5.00	16.00	-0.80	0.02	-4.59	0.01	-1.76	245.82	14
5-Phenylvaleric acid-4-sulfate-4'-glucuronide	466.41	6.23	1238.51	5.00	16.00	-0.88	0.01	-4.49	0.01	-1.76	246.71	14
4-Hydroxy-5-phenylvaleric acid	194.23	3.70	717.76	2.00	3.70	1.87	118.82	-1.02	62.93	-0.56	70.74	6
5-Phenylvaleric acid-4-glucuronide	370.36	6.48	1108.77	4.00	11.50	0.49	0.81	-2.97	0.37	-1.14	176.72	11
5-Phenylvaleric acid-4-sulfate	274.29	2.45	830.27	2.00	6.00	1.59	4.01	-1.97	2.11	-0.86	120.85	8
5-(3',4'-Dihydroxyphenyl)valeric acid	210.23	1.53	748.43	3.00	3.50	1.36	25.95	-1.74	12.15	-0.59	94.73	7
5-(4'-Hydroxyphenyl)valeric acid-3'-sulfate	290.29	4.98	872.53	3.00	7.25	0.80	1.27	-2.67	0.61	-1.01	140.53	9
5-(3'-Hydroxyphenyl)valeric acid-4'-sulfate	290.29	2.93	873.02	3.00	7.25	0.80	1.24	-2.68	0.60	-1.01	140.83	9
5-(4'-Hydroxyphenyl)valeric acid-3-glucuronide	386.36	3.69	1154.50	5.00	11.30	0.07	0.18	-3.98	0.07	-1.15	199.34	12

5-(3'-Hydroxyphenyl)valeric acid-4-glucuronide	386.36	5.08	1147.91	5.00	11.30	0.15	0.25	-3.74	0.10	-1.13	199.22	12

Legend. MW: molecular weight; Dipole: dipole moment for the molecule; Volume: volume of the molecule; donorHB: number of hydrogen bounds donor atoms; accptHB: number of hydrogen bounds acceptor atoms; QPlogPo/w: logarithm of octanol/water partition coefficient; QlogBB: logarithm of BBB predicted partition coefficient; QPPCaco: predicted permeability for Caco-2 cell line; QPPMDCK: predicted permeability for MDCK cell line; QPlogKhsa: prediction logarithm of albumin binding; PSA: Van der Waals surface area of polar atoms; #rotor: number of rotatable bounds.
